# Isolation and functional characterization of a cDNA coding a hydroxycinnamoyltransferase involved in phenylpropanoid biosynthesis in *Cynara cardunculus *L

**DOI:** 10.1186/1471-2229-7-14

**Published:** 2007-03-20

**Authors:** Cinzia Comino, Sergio Lanteri, Ezio Portis, Alberto Acquadro, Annalisa Romani, Alain Hehn, Romain Larbat, Frédéric Bourgaud

**Affiliations:** 1 Di.Va.P.R.A. Plant Genetics and Breeding, University of Torino 10095, Grugliasco (Turin), Italy; 2 Department of Pharmaceutical Sciences, University of Florence, 50019, Sesto Fiorentino (Florence), Italy; 3 UMR 1121 INPL-INRA Agronomie Environnement, 54505 Vandoeuvre-lès-Nancy, France

## Abstract

**Background:**

*Cynara cardunculus *L. is an edible plant of pharmaceutical interest, in particular with respect to the polyphenolic content of its leaves. It includes three taxa: globe artichoke, cultivated cardoon, and wild cardoon. The dominating phenolics are the di-caffeoylquinic acids (such as cynarin), which are largely restricted to *Cynara *species, along with their precursor, chlorogenic acid (CGA). The scope of this study is to better understand CGA synthesis in this plant.

**Results:**

A gene sequence encoding a hydroxycinnamoyltransferase (HCT) involved in the synthesis of CGA, was identified. Isolation of the gene sequence was achieved by using a PCR strategy with degenerated primers targeted to conserved regions of orthologous HCT sequences available. We have isolated a 717 bp cDNA which shares 84% aminoacid identity and 92% similarity with a tobacco gene responsible for the biosynthesis of CGA from *p*-coumaroyl-CoA and quinic acid. *In silico *studies revealed the globe artichoke HCT sequence clustering with one of the main acyltransferase groups (i.e. anthranilate N-hydroxycinnamoyl/benzoyltransferase). Heterologous expression of the full length HCT (GenBank accession DQ104740) cDNA in *E. coli *demonstrated that the recombinant enzyme efficiently synthesizes both chlorogenic acid and *p*-coumaroyl quinate from quinic acid and caffeoyl-CoA or *p*-coumaroyl-CoA, respectively, confirming its identity as a hydroxycinnamoyl-CoA: quinate HCT. Variable levels of HCT expression were shown among wild and cultivated forms of *C. cardunculus *subspecies. The level of expression was correlated with CGA content.

**Conclusion:**

The data support the predicted involvement of the *Cynara cardunculus *HCT in the biosynthesis of CGA before and/or after the hydroxylation step of hydroxycinnamoyl esters.

## Background

*Cynara cardunculus *L. is a perennial member of the *Asteraceae *family and has been sub-classified into three taxa: globe artichoke (var. *scolymus *L.), cultivated cardoon (var. *altilis *DC), and wild cardoon [var. *sylvestris *(Lamk) Fiori]. Molecular, cytogenetic and isozyme evidence suggests that wild cardoon is the ancestor of both cultivated forms [1-3].

Globe artichoke plays an important role in human nutrition, mainly in Mediterranean diet: immature inflorescences (capitula), commonly referred to as 'heads' or 'buds', are consumed as a fresh, canned or frozen vegetable, while more recently, demand has been driven by its reputation as health food.

The species has interesting applications in pharmacology [4]. The roots contain inulin, a natural fibre found to improve the balance of beneficial bacteria in the human gut, while the leaves and heads represent natural sources of bioactives such as luteolin and mono- and di-caffeoylquinic acids [5-8]. These compounds have been implicated in (i) the protection of proteins, lipids and DNA from oxidative damage caused by free radicals [9-11], (ii) the inhibition of cholesterol biosynthesis, contributing to the prevention of arteriosclerosis and other vascular disorders [10,12,13], (iii) hepatoprotective, choleretic, diuretic and bile-expelling activities [4], (iv) the inhibition of HIV integrase, a key player in HIV replication and its insertion into host DNA [14,15], and (v) antibacterial and antifungal activities [16-18]. All of these bioactivities have been attributed to the phenolics in the phenylpropanoid pathway [7], which is plant-specific [19]. The pathway catalyses the conversion of phenylalanine into a variety of hydroxycinnamic acids, which are the precursors for flavonoids, hydroxycinnamic acid conjugates and lignins [20]. Among phenolics, mono- and di-caffeoylquinic acids play a key-role in the overall anti-oxidant/health value of globe artichoke [8]. However, no information is available yet about the synthesis of these compounds in *C. cardunculus*.

This paper describes the cloning and biochemical characterization of an acyltransferase cDNA from globe artichoke. We explore the relationship between acyltransferases transcription and polyphenolic content in leaves, and establish a positive correlation between acyltransferase expression in various *C. cardunculus *accessions and polyphenolic content, especially CGA. Our observations suggest this acyltransferase is implicated in the biosynthesis of CGA and its derivatives.

## Results

### Isolation of acyltransferase gene in globe artichoke

**Figure 1:**
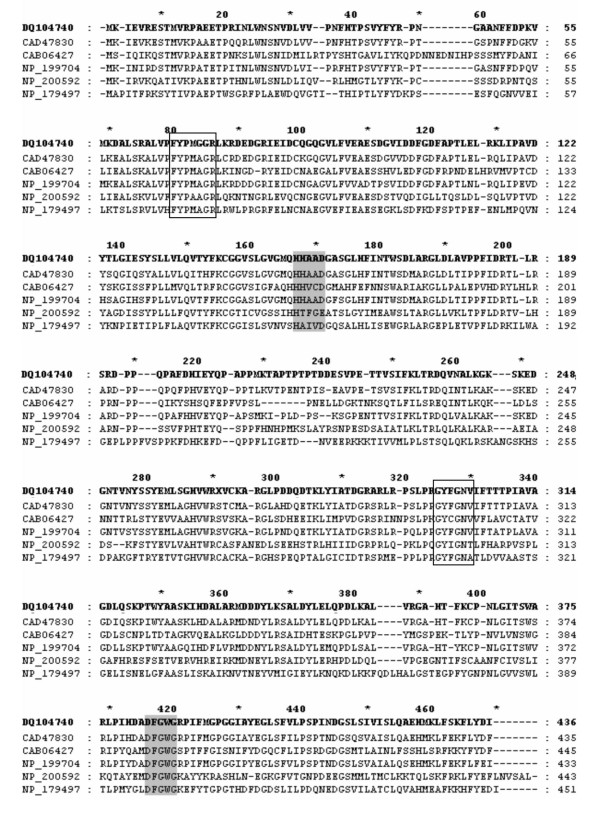
**Sequence alignment of HCT from artichoke with representative members of the plant hydroxycinnamoyl transferase family**. CAD47830 from *N. tabacum*; NP_199704 from *A. thaliana*; CAB06427 from *D. caryophyllus*; NP_200592 from *A. thaliana*; NP_179497 from *A. thaliana*; DQ104740 (in bold) from *C. cardunculus*. Black frames indicate regions chosen to design CODEHOP; gray boxes indicate structural motifs conserved in the acyltransferase family.

**Figure 2:**
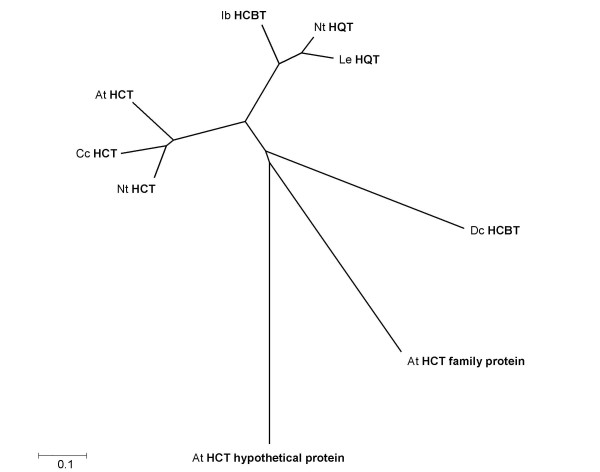
**Phylogenetic analysis of acyltransferases**. The tree was constructed by the neighbour-joining method. The length of the lines indicates the relative distances between nodes. Protein sequences used for the alignment are: DcHCBT, anthranilate N-hydroxycinnamoyl/benzoyltransferase of *D. caryophyllus *(Z84383); IbHCBT, N-hydroxycinnamoyl/benzoyltransferase from *I. batatas *(AB035183); AtHCT, shikimate/quinate hydroxycinnamoyltransferase of *A. thaliana *(At5g48930); NtHCT, shikimate/quinate hydroxycinnamoyltransferase of *N. tabacum *(AJ507825); NtHQT, hydroxycinnamoyl CoA quinate transferase of *N. tabacum *(CAE46932); LeHQT, hydroxycinnamoyl CoA quinate transferase of *L. esculentum *(CAE46933); At2G19070 and At5G57840, *A. thaliana *genes encoding putative acyltransferases; CcHCT, hydroxycinnamoyl CoA quinate transferase from *C. cardunculus *(DQ104740, this work).

Using CODEHOP, targeting conserved regions of acyl transferase proteins (Fig. 1, black frames; Table 1), a 717 bp incomplete globe artichoke acyltransferase sequence was amplified. This sequence was extended towards both the 3'- and 5'-ends of the gene by RACE-PCR and a 1480 bp sequence was isolated. The resulting 1308 bp ORF (GenBank accession DQ104740) encodes a 436-residue protein (Fig. 1 in bold) with a predicted molecular weight of ~50 kDa. Its closest *in silico *match is with a tobacco shikimate/quinate HCT [21], with which it shares 84% identity and 92% similarity; both proteins belong to a multifunctional superfamily of plant acyltransferases [22]. The globe artichoke acyltransferase has a histidine-containing motif (HHAAD, aa 153–157, Fig. 1, gray boxes) identical to the highly conserved motif HXXXD which is characteristic for acyl transfer catalysis. A second consensus sequence, the DFGWG block found in other acyltransferases [22-24], is present from aa 382 to 386 (Fig. 1, gray boxes). The next most closely related sequence to the globe artichoke acyltransferase is an *Arabidopsis thaliana *HCT (80% identity and 89% similarity) (Fig. 2). More distantly related acyltransferases are those annotated as hydroxycinnamoyl-CoA quinate: hydroxycinnamoyl transferase (HQT) from tobacco and tomato [25].

### Heterologous expression of the identified acyltransferase

**Figure 3:**
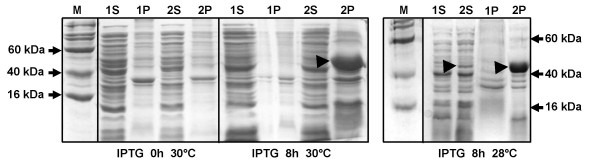
**Expression of recombinant HCT in *E. coli***. Protein content of non-induced (T = 0 h) and induced (T = 8 h) non-transformed cells (1) were compared with those of non-induced (T = 0 h) and induced (T = 8 h) transformed cells (2). After induction at 37°C for 8 h, HCT protein cannot be detected in the soluble fraction (2 S at T = 8 h), but is present in the soluble fraction after induction at 28°C. Arrows indicate the ~50 kDa HCT protein. Molecular marker masses indicated on the left. (1 = empty vector; 2 = vector -HCT; S = soluble fraction; P = pellet; M = molecular weight marker).

To assess the activity of the globe artichoke isolated acyltransferase, the cDNA was cloned and heterologously expressed in *E. coli*, through the expression vector pET3a. The recombinant plasmid or the void pET3a vector (control) were introduced into *E. coli *BL21 (DE3) pLysE cells, and expression of soluble acyltransferase was first tested at standard induction temperature (37°C for 8 h). SDS-PAGE analysis indicated that the pellet fraction of recombinant bacteria contained an overexpressed protein with an apparent molecular mass of approximately 50 kDa, consistent with the expected mass for the translation product of the acyltransferase cDNA (Fig. 3). No acyltransferase protein could be detected by SDS-PAGE in the supernatant fraction of lysed recombinant cells. Reducing the temperature to 28°C during IPTG induction for 8 hours allows to increase the amount of soluble recombinant enzyme produced (Fig. 3) which can be detected in both fraction: pellet and supernatant. No corresponding protein is observed in samples prepared from bacteria carrying the control void vector.

### Enzyme assay

**Figure 4:**
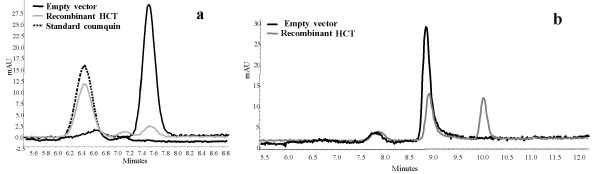
**HPLC analysis of the HCT reaction products**. An aliquot of the incubation reaction without (black line) or with (gray line) recombinant HCT was analysed. (a) HCT reaction with *p*-coumaroyl-CoA and quinate; standard of *p*-coumaroyl-quinate (dotted line) is used as reaction control; (b) HCT reverse reaction with chlorogenic acid and CoA.

**Figure 5:**
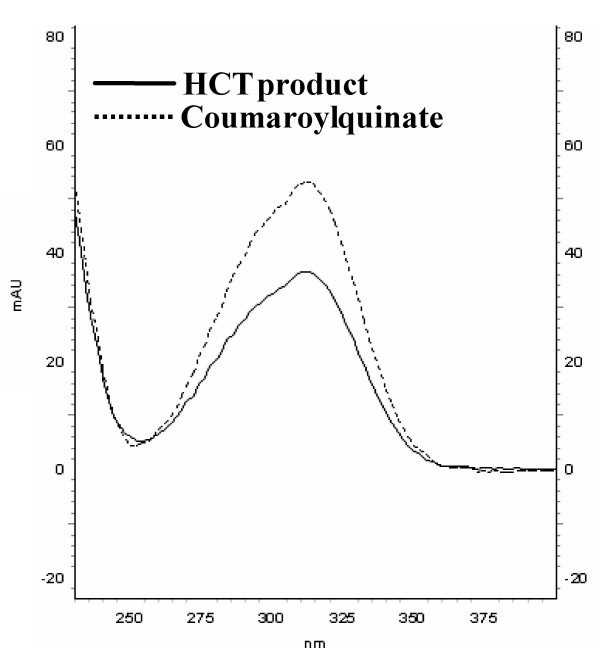
**Example of comparison between absorption spectrum of the reaction product and authentic standard**. Absorption spectra of *p*-coumaroyl-quinate: standard (dotted line) and product by reaction with HCT (black line).

The recombinant acyltransferase was used for substrate specificity studies. HPLC profiles were generated after incubation of the substrates caffeoyl-CoA or *p*-coumaroyl-CoA, with quinate or shikimate in the presence of recombinant bacteria crude extract carrying the pET-HCT or the void control vector. Both caffeoyl-CoA and *p*-coumaroyl-CoA were accepted as substrates, with either quinate and shikimate, to synthesize caffeoylquinate (i.e. chlorogenic acid), *p*-coumaroyl quinate, caffeoylshikimate or *p*-coumaroyl shikimate, depending on the substrates supplied. In presence of the recombinant proteins, a new product was detected in all cases (Fig. 4, grey lines); new peaks could not be detected when the reaction was performed with the control crude extract (black lines in Fig. 4). The reaction product was identified by comparing its retention time and its absorbance spectrum (200–400 nm, Fig. 5). We also successfully investigated the ability of the isolated acyltransferases to catalyse the reverse reaction (*i.e*. production of caffeoyl-CoA from chlorogenic acid), as described in other species [21,26,27]: caffeoyl-CoA was detected when the chlorogenate was incubated with CoEnzyme A in presence of the recombinant protein (Fig. 4b).

### Determination of kinetic parameters

Kinetic parameters of the HCT enzyme were evaluated for the different substrates (Table 2). The affinity of the enzyme for quinate as acceptor was higher than for shikimate. Moreover *p*-coumaroyl-CoA was the most efficient donor, with a *V*_max_/*K*_m _of 0.041, followed by caffeoyl-CoA, with a value of 0.01.

### Identification and quantification of polyphenolics

**Figure 6:**
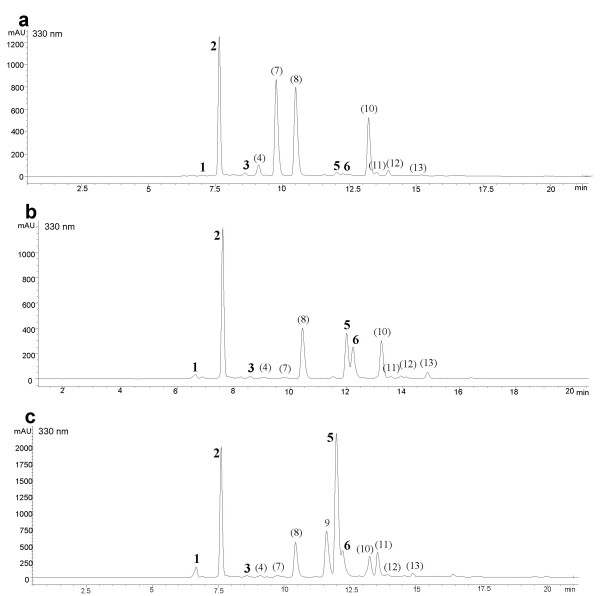
**HPLC/DAD profiles at 330 nm of the compounds identified in *C. cardunculus***. (a) globe artichoke, (b) cultivated cardoon, and (c) wild cardoon. Peaks: 1. caffeoylquinic acid; 2. chlorogenic acid (5-CQA); 3. *p*-coumaroylquinic acid; 4. feruloylquinic acid; 5. and 6. di-caffeoylquinic acids; 7. luteolin 7-O-rutinoside; 8. luteolin 7-O-glucoside; 9. luteolin 7-O-glucuronide; 10. luteolin malonylglucoside; 11. apigenin 7-O-glucuronide; 12. luteolin; 13. apigenin. In bold are indicated the caffeoylquinic acids and in brackets others compounds detected in *C.cardunculus*.

Six compounds belonging to quinic acid esters were quantified (Table 3, see Fig. 6 for chromatograms). 'Violet Margot' and wild cardoon leaves presented a high mean content of total caffeoylquinic acid (respectively, 60.8 ± 1.98 and 59.9 ± 4.30 mg/g dry matter) while the cultivated cardoon and 'Romanesco C3' leaves showed lower contents (17.1 ± 2.59 and 15.0 ± 3.10 mg/g dry matter). Chlorogenic acid was the most abundant compound among quinic acid esters in globe artichoke (both 'Romanesco C3' and 'Violet Margot') and cultivated cardoon, but was less represented than di-caffeoylquinic acids in wild cardoon. All samples contained very low levels of *p*-coumaroylquinic acid.

### Northern blot analysis

**Figure 7:**
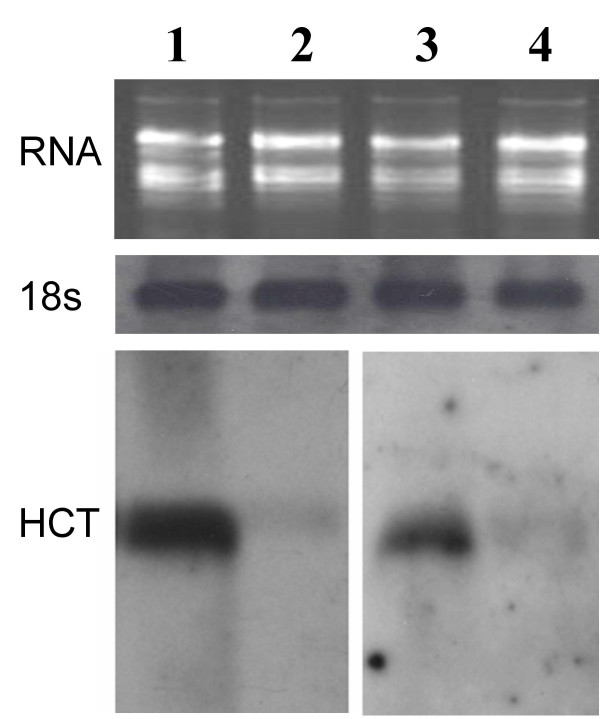
**Northern blot analyses for HCT expression**. First and second panels are, respectively, total RNA ethidium bromide-stained prior to membrane transfer and 18S expression to control RNA quality and sample loading. Third panel is the HCT expression with probe1 in 'Violet Margot' (1), Romanesco C3' (2), and with probe 2 in wild cardoon (3) and cultivated cardoon (4).

A northern blot approach was taken to identify a possible correlation between HCT expression and polyphenolics/chlorogenic acid content. As the different plant species probably not exhibit exactly the same HCT sequence, PCR was performed on each genomic DNA in order to isolate a highly specific HCT probe (probe 1 from globe artichoke, probe 2 from cultivated cardoon, and probe 3 from wild cardoon). A sequence alignment established a high level of similarity (99%) between probe 2 and 3 (respectively isolated from cultivated cardoon and wild cardoon), but a rather lower level (80%) between probes 2 (or 3) and probe 1, from globe artichoke (data not reported). When 'Romanesco C3' and 'Violet Margot' RNA were probed with the globe artichoke HCT sequence (probe 1), the latter showed a higher transcript abundance in its leaves than the former (Fig. 7). Moreover, northern blot on cultivated and wild cardoon RNAs challenged with either probe 2 and 3, showing that a higher level of HCT transcript was present in the wild form (Fig. 7). Results are summarized in table 3.

## Discussion

**Figure 8:**
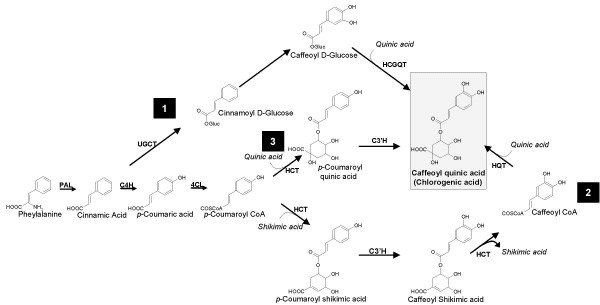
**The three routes in phenylpropanoid metabolism (labelled 1, 2 and 3) proposed for the chlorogenic acid synthesis in plants**. Enzymes involved in the pathway are: PAL, phenylalanine ammonia lyase; C4H, cinnamate 4-hydroxylase; 4CL, 4-hydroxycinnamoyl CoA ligase; HCT, hydroxycinnamoyl CoA shikimate/quinate hydroxycinnamoyl transferase; C3H, p-coumarate 3'-hydroxylase; HQT, hydroxycinnamoyl CoA quinate hydroxycinnamoyl transferase; UGCT, UDP glucose: cinnamate glucosyl transferase; HCGQT, hydroxycinnamoyl D-glucose: quinate hydroxycinnamoyl transferase (adapted from [25]).

Phenolic compounds are by far the commonest of plant therapeutic molecules [28], and the major species present in globe artichoke leaves are the di-caffeoylquinic acids (e.g. cynarin), and their precursor CGA, a soluble phenolic which is widespread throughout the plant kingdom. The definition of the CGA biosynthetic pathway remains controversial, with three alternative routes (Fig. 8) under current consideration [25]. These are (1) CGA synthesis using a caffeoyl-glucoside as the active intermediate; (2) synthesis of CGA from caffeoyl-CoA and quinic acid by means of HQT (hydroxycinnamoyl-CoA: quinate HCT), which differs from HCT in its preference for quinate over shikimate as a substrate; and (3) synthesis of *p*-coumaroyl-quinate by HCT or HQT and its subsequent hydroxylation by *p*-coumarate-3'-hydroxylase (C3'H) to form CGA. The first route has been identified in sweet potato by Villegas and Kojima [29], who were able to purify hydroxycinnamoyl D-glucose:quinate HCT and show that caffeoyl D-glucose and quinic acid are the substrates for the biosynthesis of CGA. Routes (2) and (3) were unequivocally established by Ulbrich and Zenk in several differentiated plants and undifferentiated cell suspension cultures [27].

Recently, both the second and third CGA synthesis routes have received experimental support. The biochemical characterization of C3'H [30,31] and hydroxycinnamoyl-CoA transferase HCT [21] suggests that CGA can be synthesized via the third route. However, since both HCT and C3'H are active in *A. thaliana*, a species which does not accumulate CGA, it is unlikely that this route can be generally exploited by plants which accumulate significant amounts of CGA [25]. In tomato, it was difficult to establish whether HQT acts directly on caffeoyl-CoA and quinic acid to produce CGA, or whether it synthesizes *p*-coumaroyl quinate from *p*-coumaroyl-CoA and quinic acid, which is subsequently converted to CGA by the activity of C3'H [25]. The second route was assumed to be dependent on the relative sizes of the caffeoyl-CoA and *p*-coumaroyl-CoA pools present. Nevertheless, strong support for the prevalence of the second route, at least in tomato, was provided by experiments in which the silencing of HQT caused the level of leaf CGA to fall by 98%, and to rise by 85% when it was over-expressed.

In a study of the phenolic content in various globe artichoke tissues and organs, total phenol concentration was shown to be greatest in the leaves, and declined in the heads during their development [8]. The variation in antioxidant activity (generated by phenolic compounds) in globe artichoke extracts may, therefore, be attributed to the choice of plant tissue used as the source of extract, rather than to any variation in genotype or environment. Thus we used leaf as our source of mRNA in order to generate the necessary cDNA, and exploited CODEHOP to isolate globe artichoke HCT. The heterologous (in *E. coli*) expression product of the cloned HCT sequence was a ~50 kDa recombinant protein, which was active when provided with either *p*-coumaroyl-CoA or caffeoyl-CoA ester as acyl donors, at comparable *K*_m _values of 53.0 ± 13.0 μM and 61.7 ± 0.004 μM, respectively. Moreover, the artichoke HCT showed a preference for quinic acid over shikimic acid as an acceptor (53.0 ± 13.0 μM *vs *701.7 ± 52.0 μM). This behaviour contrasts with that of tobacco HCT [21], but is consistent with the activity of HQT isolated from tobacco and tomato [25]. Interestingly, although the globe artichoke HCT sequence is closely related to that of its tobacco ortholog, its activity appears to be more similar to that of tobacco and tomato HQT.

In order to evaluate the role of HCT in the biosynthetic pathway of CGA in globe artichoke, the purified enzyme was provided *in vitro *with quinic acid and either *p*-coumaroyl-CoA or caffeoyl-CoA. Since the enzyme was active with both *p*-coumaroyl-CoA and caffeoyl-CoA, it is clear that this HCT can act either before and/or after 3'-hydroxylation step. Other experiments have demonstrated that the heterologously expressed HCT, in the presence of quinic acid as the acyl donor, is four times more efficient when provided with *p*-coumaroyl-CoA rather than with caffeoyl-CoA (*V*_max_/*K*_m _values of, respectively, 0.041 and 0.01). However, these observations do not constitute an absolute proof that the third biosynthetic route is preferred over the second, since the level of HCT is not necessarily limiting *in vivo*. Note that CGA synthesis is also regulated by its interaction with C3'H, a P450 whose enzymatic turnover was found to be low [32].

Globe artichoke HCT belongs to a versatile plant acyltransferase family that shares certain structural motifs (Fig. 1, grey boxes), including several plant members involved in a number of secondary metabolism pathways. When the sequence alignment of the acyltransferase family was used to construct a phylogenetic tree (Fig. 2), the globe artichoke HCT was found to cluster with the major anthranilate N-hydroxycinnamoyl/benzoyltransferase group defined by Burhenne et al. [33]. It is clearly closely related to its tobacco and *A. thaliana *orthologs.

*C. cardunculus *includes two crop species, the globe artichoke and the cultivated cardoon, along with the ancestral wild cardoon. In our samples, *p*-coumaroylquinic acid was ubiquitously detected at a low concentration (Table 3), presumably because this quinate ester is a transient intermediate, unlike chlorogenic acid, which is considered to be an accumulation product in several plant species [30]. Di-caffeoylquinic acid synthesis remains unknown in higher plants. However, due to their close structural relationship with CGA, it is reasonable to suppose that the di-caffeoylquinic acids are derived from simple quinic acid monoesters. CGA and di-caffeoylquinic acid quantification studies on the leaves of four plant accessions were carried out to identify any correlations between these two families of molecule (Table 3). The globe artichoke 'Violet Margot' and the cultivated cardoon contained comparable levels of CGA and di-caffeoylquinic acids. On the other hand, in the globe artichoke 'Romanesco C3' there was ten fold more CGA than di-caffeoylquinic acids, while in the wild cardoon, this difference was about two fold. Therefore, the regulation of the synthesis of di-caffeoylquinic acids should be, possibly, genotype-dependent.

Northern blots, using cDNA from the same three *C. cardunculus *subspecies analysed above, were performed to study patterns of HCT expression. As these diverse genotypes could carry distinct allelic forms of HCT, we developed three species-specific probes [probes 1 (globe artichoke), 2 (cultivated cardoon) and 3 (wild cardoon). A positive relationship between the quantity of HCT transcript and the content of caffeoylquinic acids was observed in all accessions. Since HCT silencing induces an increase (or no significant change) in the amount of caffeoylquinic compounds in tobacco [34], whereas HQT silencing (in tomato) results in a decrease in CGA content [25], HQT transcripts may well play a pivotal role in determining the make-up of the CGA pool, and the behaviour of HCT in globe artichoke is fully consistent with this model.

## Conclusion

CGA is particularly abundant in species belonging to the *Asteracaeae*, *Solanaceae *and *Rubiaceae *families [35], but its mode of biosynthesis is still unclear in many plants. We have described the cloning and expression of HCT, an acyltransferase acting both upstream and downstream of the 3'-hydroxylation step. In addition, for both wild and cultivated forms of *C. cardunculus*, the expression of HCT appears to be correlated with leaf polyphenolic content, especially with respect to caffeoylquinic acid derivatives, suggesting that this HCT has an essential role in the synthesis of CGA and related esters.

In a recent report [25], caffeoyl-CoA has been firmly established as a major substrate for the acylation of quinic acid and the synthesis of CGA in Solanaceous plants. Our future research activity will be focused in analysing the *in vivo *expression of HCT, as well as on the isolation of other acyltransferases, such as HQT, which may be involved in the phenylpropanoid pathway of *C. cardunculus*

## Methods

### Plant material and RNA extraction

Leaves of globe artichoke, cultivated cardoon and wild cardoon were collected from experimental fields at the University of Catania in Cassibile, Sicily (Italy). Total RNA was extracted from approximately 100 mg fresh tissue using the "RNAwiz" reagent (Ambion, USA), following the manufacturer's instructions. Final RNA concentration was determined by spectrophotometry, and its integrity was assessed by electrophoresis in 1% (w/v) formaldehyde-agarose gel [36].

### Purification and cloning of globe artichoke HCT

Reverse transcription from total RNA was achieved using poly(dT)primer and M-MuLV RNaseH^- ^RT (Finnzymes, Finland), following the manufacturer's instructions. Incomplete cDNAs were derived by PCR, using as template the cDNA generated by reverse transcription. Based on conserved regions of the acyltransferase amino acid sequence (Fig. 1), primers COD1For and COD1Rev (Table 1) were designed, with each primer consisting of a short 3' degenerate core and a longer 5' consensus clamp region. As recommended by Morant et al. [37], a cDNA amplification step was first performed, and the fragment of expected size was isolated from 1% agarose gel separations of the total amplicon. DNA sequences were resolved by BMR genomics [38]. Specific primers were designed for 3'- and 5'-end amplification of the HCT transcript, based on the derived incomplete cDNA sequence (Table 1). For the 3'-end, the template was the poly(dT) reverse transcription product, and the primers consisted of poly(dT) oligonucleotides in combination with the specific primers ART2For and ART2For-nested (Table 1). The fragment of expected size was isolated from an agarose gel separation, cloned into pCR^®^2.1 (Invitrogen, USA), and sequenced. For the 5'-end, full-length cDNA produced with the SuperScript™ Plasmid System (Invitrogen, USA) was inserted into the pCMV•SPORT6 plasmid. PCR was performed on this cDNA library using the antisense primer ART2Rev and ART2Rev-nested (Table 1), along with the universal SP6 specific primer. The expected fragment was isolated from an agarose gel separation, cloned into pCR^®^2.1 (Invitrogen, USA) and sequenced. The full length sequence was validated by an amplification using primers UTR5' and UTR3' (Table 1), designed from the sequence of the 5'- and 3'-ends of the HCT transcript. Multiple sequence alignments were performed using ClustalW [39], with standard parameters. Phylogenetic analysis was conducted using MEGA version 3.0 [40].

### Heterologous expression in *E. coli*

The HCT open reading frame (ORF) was amplified using HCTFor and HCTRev primers (Table 1), which additional recognition sites *Nde*I (5'-end) and *Bam*HI (3'-end). The amplified fragment was restricted with *Nde*I and *Bam*HI, and ligated into the cloning site of digested pET3a plasmid (Novagen, USA). The resulting recombinant pET3a-HCT plasmid was transformed into *E. coli *strain BL21(DE)pLysE, and grown on a selective medium (LB, Luria-Bertani, broth agar with 50 mg/l ampicillin and 34 mg/l chloramphenicol). Individual colonies were transferred to 4 ml overnight cultures in the same medium, and 2 ml of bacterial cell suspensions were then grown for 3 h at 28°C in 50 ml of LB liquid medium with shaking (200 rpm). Isopropyl-β-D-thiogalactopyranoside (IPTG) was added to a final concentration of 1 mM and the cultures were grown for further 8 h at 28°C. After centrifugation for 10 min at 5000 g, the pellet was resuspended in 1 ml of phosphate-buffered saline pH 7.5 and lysed by three cycles of freezing (in liquid nitrogen) and thawing (at 37°C), followed by three bursts of 30 s sonication on ice. Sonicated cells were centrifuged at 4°C and 14,000 g for 5 min, and both supernatant and pellet were assayed for HCT activity, and profiled by SDS-PAGE (10% resolving gel, 5% stacking gel) using Coomassie brilliant blue staining [36]. Negative controls used comparable preparations harbouring a void vector.

### Enzyme assay and the determination of kinetic parameters

CGA was purchased from Sigma-Aldrich (Germany), and quinic acid from Fluka (Switzerland). CoA esters (substrates) were kindly provided by Dr. Legrand (CNRS-IBMP laboratory Strasbourg, France) and then synthesised using the procedure proposed by Beuerle and Pichersky [41]. 4CL enzyme was kindly provided by Dr. Douglas (University of British Columbia Vancouver). The 20 μl reaction mixture contained 100 mM sodium phosphate buffer (pH 7.5), 1 mM dithiothreitol, between 50 ng and 1 μg of enzyme, and the various substrates (*p*-coumaroyl-CoA, caffeoyl-CoA, quinic acid and shikimic acid) at concentrations ranging from 0.1 mM to 5 mM. The reverse reaction, i.e. conversion of chlorogenic acid and CoA into caffeoyl-CoA, was tested as follow: 0.8 μg enzyme was incubated in presence of 1 mM dithiothreitol, 100 μM of chlorogenate and 100 μM CoA. Reactions were initiated by enzyme addition, incubated at 30°C for 30 min, and terminated by the addition of 20 μl of acetonitrile/HCl (99:1). Reaction products were analysed by reverse-phase HPLC on a C18 column (LiChroCART 125-4, Merck), using the pair of solvents 90% H_2_O, 9.9% CH_3_CN, 0.1% HCOOH and 80% CH_3_CN, 19.9% H_2_O, 0.1% CH_3_COOH. The percentage of the latter reached the 60% over a 15 min run time, and 100% after 28 min.

Varying substrate-dependent concentrations were used for *K*_m _determination. These consisted of 0.8 μg enzyme, 300 μM *p*-coumaroyl-CoA and 0.1–30 mM quinate for *K*_m _of quinate. *K*_m _of *p*-coumaroyl-CoA was established with 10–400 μM *p*-coumaroyl-CoA with quinate (20 mM) or 100–600 μM with shikimate (20 mM) as acceptors and 0.8 μg enzyme. For caffeoyl-CoA *K*_m _determination, 0.8 μg enzyme, 20 mM quinate and 10–100 μM caffeoyl-CoA were used. Reactions were incubated at 30°C for 10 min. An HPLC calibration curve was established for each molecule for quantification purpose. *K*_m _and *V*_max _values were calculated from triplicated by Lineweaver-Burk plots.

### Extraction of polyphenolic compounds, HPLC/DAD and HPLC/MS analyses

On the basis of a genetic diversity assessment of 118 globe artichoke accessions [2] about 30 entries were chosen to represent the genetic variation present. Leaves of these entries were assayed in triplicate for leaf polyphenolic composition using HPLC/DAD/MS. Mono- and di-caffeoylquinic derivatives, apigenin and luteolin glycosides were characterised and quantified. On the basis of these observations, two globe artichoke entries ('Romanesco C3' and 'Violet Margot'), one cultivated cardoon (accession '41') and one wild cardoon (accession 'Kamaryna') were carried forward for a detailed study of the correlation between leaf polyphenolic composition and HCT expression.

Leaf tissues from these four different plants at 22–23 fully-expanded leaves stage were collected. Growth conditions and management practices were similar to those used in commercial cultivation. Half of the plant material has been freezed immediately in liquid nitrogen, whereas the other part was lyophylized within 12 h of collection and the dried materials were ground to a powder and stored in sealed bags at room temperature. The powders (2 g) were extracted in 70% v/v ethanol (pH 2), cleared through a 0.45 μm filter, and the extractants first concentrated under vacuum (Rotavapor 144 R, Büchi, Switzerland) then rinsed with ultra pure acid water (MilliQ system, Waters Co., USA) adjusted to pH 2 with formic acid. Defatting was achieved by the addition of n-hexane, and the samples were re-concentrated under vacuum to a final volume of 25 ml. Lyophilised extracts were stored at -20°C.

Leaves polyphenolic composition were analysed using HPLC/DAD and HPLC/MS. HPLC/DAD analyses were performed via a HP-1100 liquid chromatograph equipped with a DAD detector and a HP 1100 MSD API-electrospray (Agilent Technologies, USA). Polyphenol compounds were separated using a 150 × 4.6 mm (5 μm) Luna RP18 (Phenomenex) maintained at 27°C. The eluents were H_2_O (pH 3.2 adjusted with formic acid), and CH_3_CN. A four-step linear solvent gradient system was used, starting from 0% and reaching 100% CH_3_CN over a 30 min run, using a flow rate of 0.6 ml/min. The percentage of CH_3_CN was 20% from 0–5 min, 30% from 7–13 min, and 100% from 20–30 min. UV-vis spectra were recorded in the range 190–600 nm, and chromatograms were obtained at 330 nm.

HPLC/MS analyses were performed using a HP 1100L liquid chromatograph linked to a HP 1100 MSD mass spectrometer with an API/ESI interface (Agilent Technologies, California, USA). The mass spectrometer operating conditions were: gas temperature, 350°C; nitrogen flow rate, 10.5 L/min; nebulizer pressure, 40 psi; quadrupole temperature, 40°C; and capillary voltage, 3500 V. The orthogonal position of the nebulizer with respect to the capillary inlet enabled the use of the same conditions as for HPLC-DAD analysis. The mass spectrometer was operated in negative mode; and the fragmentor was set at 120 eV for the standard and the isolated compounds.

Individual polyphenols were identified by comparison and combination of their retention time, UV-Vis and mass spectra of the peaks with those of authentic samples or isolated compounds previously characterized [42]. Cynarin was obtained from Roth (Germany) and chlorogenic acid from Sigma-Aldrich (St. Louis, USA). Quantification of individual polyphenols was performed directly by HPLC-DAD using a four-point regression built with the available standards. Curves with a correlation factor above 0.9998 were considered to be significant. Quantification of caffeoylquinic mono- and di-esters was inferred from 330 nm chromatograms using CGA and cynarin for reference.

### Development of *C. cardunculus *probes and northern blot analysis

For northern blot analyses, 3 probes were developed by applying a PCR Dig Probe Synthesis Kit (Roche Applied Science, Switzerland) with ART3For and ART3Rev primers (Table 1) designed from the *C. cardunculus *HCT sequence: probe 1 from globe artichoke, probe 2 from cultivated cardoon, and probe 3 from wild cardoon.

RNA were extracted from the second freezed part of the plant material described above. Total RNA separated on formaldehyde-agarose gels [36] before blotting to a nylon membrane (Amersham Pharmacia Biotech, UK) *via *over-night capillarity transfer. RNAs quality was confirmed by ethidium bromide staining of the gel (Fig. 7, first panel) following electrophoresis. Furthermore, RNA analysis was normalized by using a constitutively expressed gene (RNA 18S, Fig. 7, second panel). Hybridization was performed at 50°C for 16 h with a denatured DNA probe. After hybridization, stringency washes (2 × 15 min at RT in 2× SSC, 0.1% SDS and 2 × 15 min at 68°C in 0.1× SSC, 0.1% SDS) was performed. The membrane subsequently was incubated with anti-DIG-AP conjugate (diluted 1:20,000), and visualised using CDPStar reagents (Roche Applied Science, Switzerland) by a 15–60 min exposure to X-ray film.

## Authors' contributions

SL and FB planned and supervised the work. CC and AH carried out the molecular genetic studies; EP and AA carried out the phylogenetic analysis; AR and RL performed the HPLC's. All authors read and approved the final manuscript.

## Figures and Tables

**Table 1:** Primer sequences used to isolate HCT gene in globe artichoke

**COD1For**	5'-TTTTATCCNATGGCNGGDMG-3'
**COD1Rev**	5'-AACGTTHCCRAARTANCC-3'
**ART2For**	5'-ATGGCAACACTGTCAATTA-3'
**ART2For-nested**	5'-CCCGACGATCAGGATA-3'
**ART2Rev**	5'-ACCGCCGGGATGAGTT-3'
**ART2Rev-nested**	5'-CCGCCTCCACGAACAA-3'
**UTR5'**	5'-TTCCGTTTCGTTTCTTCAA-3'
**UTR3'**	5'-TGGCCATAACCATTTTAGATAT-3'
**HCTFor**	5'-GGGTTTCATATGAAGATCGAGGTGAGAGAA-3'
**HCTRev**	5'-CGGGATCCTTAGATATCATATAGGAACTTGC-3'
**ART3For**	5'-TCCCCAATTTTCACACAC-3'
**ART3Rev**	5'-AAGTGCCGATTTTAGATAAT-3'

**Table 2:** Kinetic parameters of recombinant HCT

**Varying substrate**	**Saturating substrate**	**kinetic parameters**		
		
		***K***_**m **_(μM)	***V***_**max **_(nkat/mg)	***V***_**max**_**/*K***_**m **_(nkat/mg/μM)
quinate	*p*-coumaroyl-CoA	9.2 × 10^3 ^± 5.6 × 10^3^	2.1 ± 1.2	0.0002
*p*-coumaroyl-CoA	quinate	53.0 ± 13.0	2.2 ± 0.5	0.041
*p*-coumaroyl-CoA	shikimate	701.67 ± 52	2.06 ± 0.55	0.003
Caffeoyl-CoA	quinate	61.75 ± 0.004	0.67 ± 0.04	0.01

The *K*_m _and *V*_max _values were calculated from triplicates by the Lineweaver-Burk method.

**Table 3:** Caffeoylquinic acids content in leaves of globe artichoke, cultivated and wild cardoon and expression level of HCT in the different plant accessions

	**Globe artichoke 'Romanesco C3'**	**Globe artichoke 'Violet Margot'**	**Cultivated cardoon**	**Wild cardoon**
Caffeoylquinic acid (1)*	0.21 ± 0.06 c	2.01 ± 0.03 a	0.61 ± 0.07 b	1.83 ± 0.03 a
**Chlorogenic acid (5-CQA) (2)**	13.49 ± 2.73 bc	32.74 ± 0.79 a	9.32 ± 1.18 c	19.02 ± 0.97 b
***p*-Coumaroylquinic acid (3)**	0.28 ± 0.03 ab	trace	0.18 ± 0.03 b	0.35 ± 0.03 a
**Feruloylquinic acid (4)**	0.96 ± 0.32 a	0.79 ± 0.06 a	0.13 ± 0.00 c	0.34 ± 0.11 b
**Dicaffeoilquinic acids (5,6)**	1.33 ± 0.66 d	26.03 ± 1.09 b	7.16 ± 1.30 c	39.04 ± 3.45 a

**Total Caffeoylquinic acids (1,2,5,6)**	**15.03 ± 3.10**	**60.78 ± 1.98**	**17.09 ± 2.59**	**59.89 ± 4.30**

**HCT expression**	**+/-**	**+**	**+/-**	**+**

Caffeoylquinic concentrations are expressed in mg/g dry matter. Within a column, means with the letter are not significantly different (P < 0.01; Tukey's HSD test).

HCT expression was measured by northern blot analysis achieved with HCT probes specifically designed for each plant accession. +/- indicates a barely detectable signal, + indicates a strong signal.

* Numbers in brackets refer to peaks reported in Fig. 6
